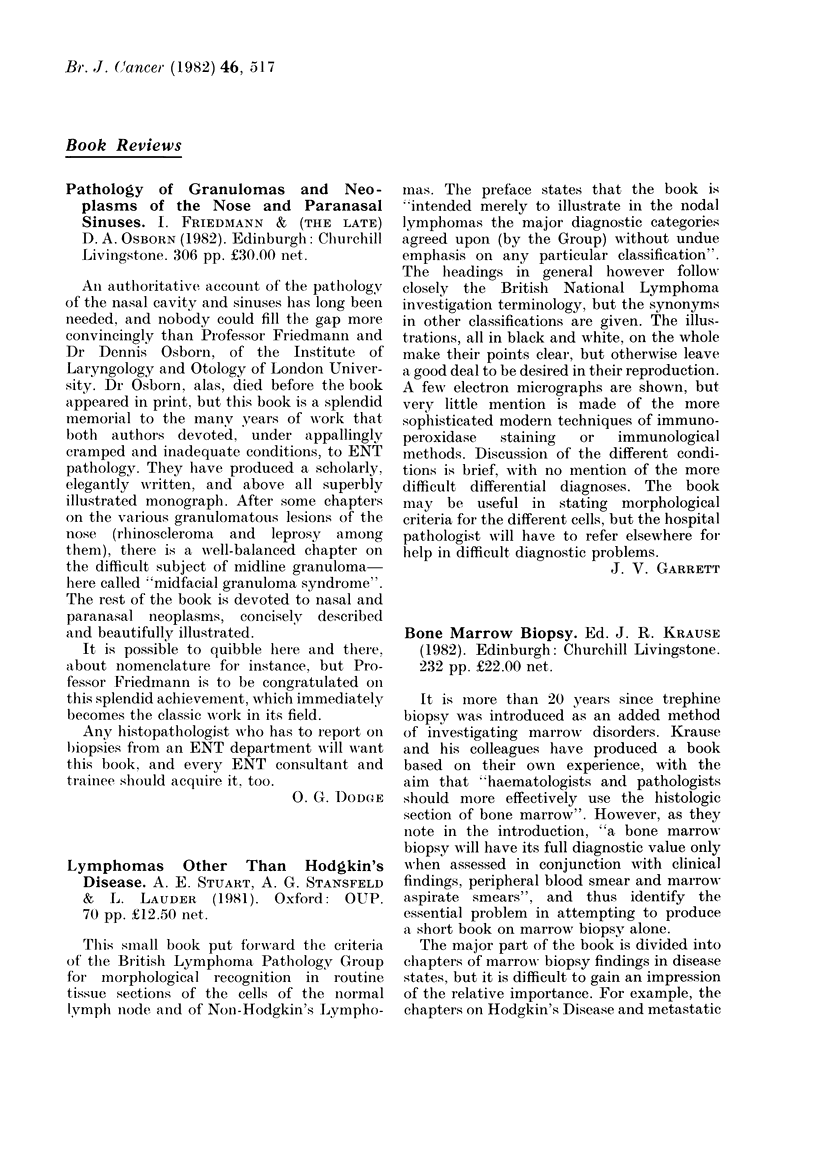# Pathology of Granulomas and Neoplasms of the Nose and Paranasal Sinuses

**Published:** 1982-09

**Authors:** O. G. Dodge


*Br. J. Cancer* (1982) **46**, 517

### *Book Reviews*

**Pathology of Granulomas and Neoplasms of the Nose and Paranasal Sinuses.** I. FRIEDMANN & (THE LATE) D. A. OSBORN (1982). Edinburgh: Churchill Livingstone. 306 pp. £30.00 net.

An authoritative account of the pathology of the nasal cavity and sinuses has long been needed, and nobody could fill the gap more convincingly than Professor Friedmann and Dr Dennis Osborn, of the Institute of Laryngology and Otology of London University. Dr Osborn, alas, died before the book appeared in print, but this book is a splendid memorial to the many years of work that both authors devoted, under appallingly cramped and inadequate conditions, to ENT pathology. They have produced a scholarly, elegantly written, and above all superbly illustrated monograph. After some chapters on the various granulomatous lesions of the nose (rhinoscleroma and leprosy among them), there is a well-balanced chapter on the difficult subject of midline granuloma—here called "midfacial granuloma syndrome". The rest of the book is devoted to nasal and paranasal neoplasms, concisely described and beautifully illustrated.

It is possible to quibble here and there, about nomenclature for instance, but Professor Friedmann is to be congratulated on this splendid achievement, which immediately becomes the classic work in its field.

Any histopathologist who has to report on biopsies from an ENT department will want this book, and every ENT consultant and trainee should acquire it, too.

O. G. DODGE